# Supplementary material for: Dynamic simulation of articulated soft robots
Source: Nat Commun. 2020 May 6;11:2233. doi: 10.1038/s41467-020-15651-9 (PMC7203284; doi:10.1038/s41467-020-15651-9)
Supplement: Supplementary file 1 — Supplementary Information [file 41467_2020_15651_MOESM1_ESM.pdf]

Supplementary Information for
Dynamic Simulation of Articulated Soft Robots

Weicheng Huang[1†], Xiaonan Huang[2†], Carmel Majidi[2*], and M. Khalid Jawed[1*]

[1]*Department of Mechanical and Aerospace Engineering, University of California, Los Angeles,*

420 Westwood Plaza, Los Angeles, CA 90095

[2]*Department of Mechanical Engineering, Carnegie Mellon University,*

5000 Forbes Avenue, Pittsburgh, PA 15213

[†] *W.H. and X.H. contributed equally to this work.*

[*]*To whom correspondence should be addressed:*

cmajidi@andrew.cmu.edu and khalidjm@seas.ucla.edu.

Supplementary Figures

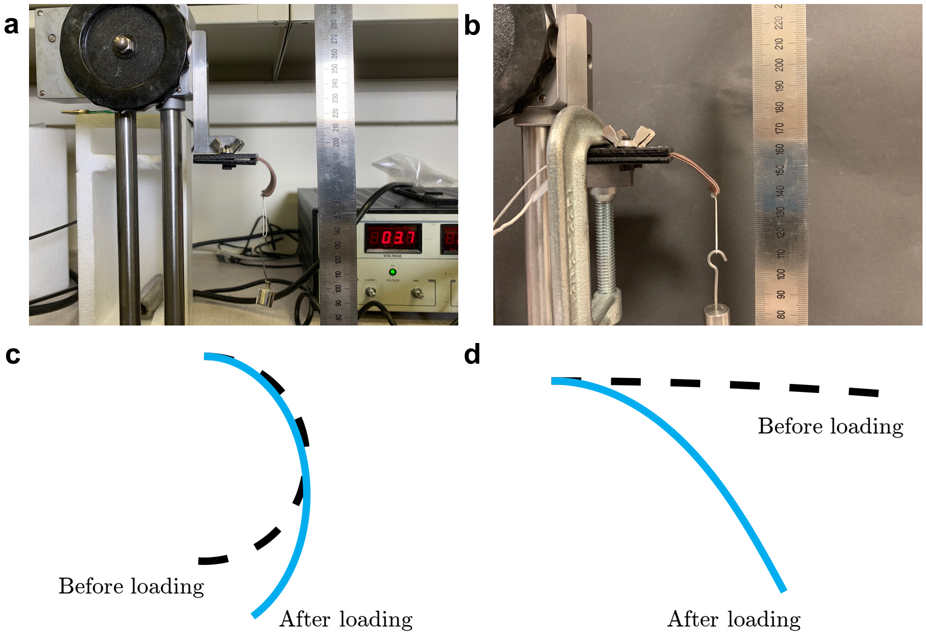

Supplementary Figure 1: Static behavior of a single shape memory alloy actuator. **a** Shape memory alloy actuator in unactuated state under loading. **b** Shape memory alloy actuator in actuated state under loading. **c** Simulation results of initial configuration (dashed line) and deformed configuration (solid line) in unactuated state. **d** Simulation results of initial configuration (dashed line) and deformed configuration (solid line) in actuated state.

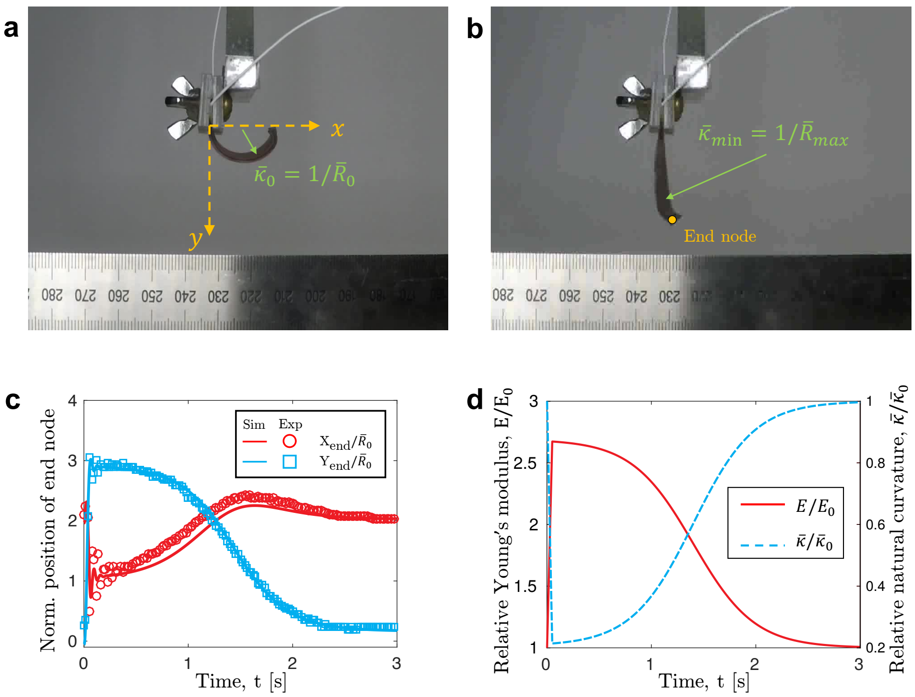

Supplementary Figure 2: Dynamic performance of a single shape memory alloy actuator. **a** The shape of SMA-based actuator before actuating. **b** Maximum response of SMA-based actuator during actuating process. **c** Relative beam end displacement $X_{\text{end}}/\bar{R}_0$ and $Y_{\text{end}}/\bar{R}_0$ from both experiments and simulations. **d** Relative Young's modulus E/E_0 (solid) and relative natural curvature $\bar{\kappa}/\bar{\kappa}_0$ (dashed) change as a function of time during actuating/cooling process.

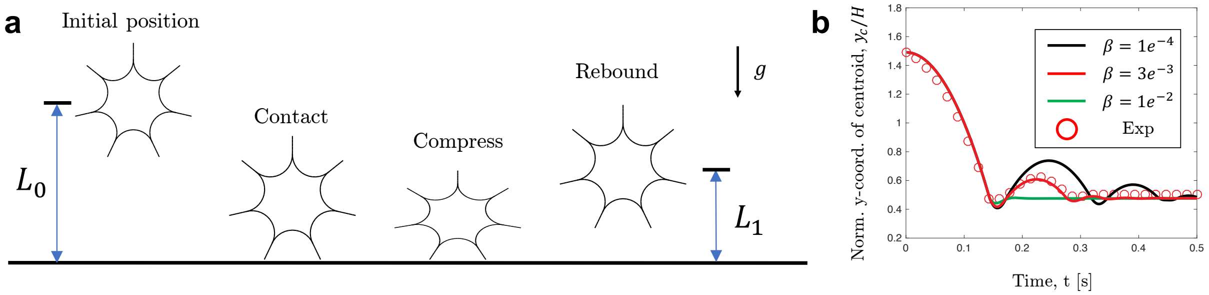

Supplementary Figure 3: Elastic/inelastic collision of soft rolling robot. **a** Collision process between rolling robot and rigid platform. **b** Normalized center position y_c/H as a function of time at different values of material damping parameter, β (solid lines) and experimental data (symbols).

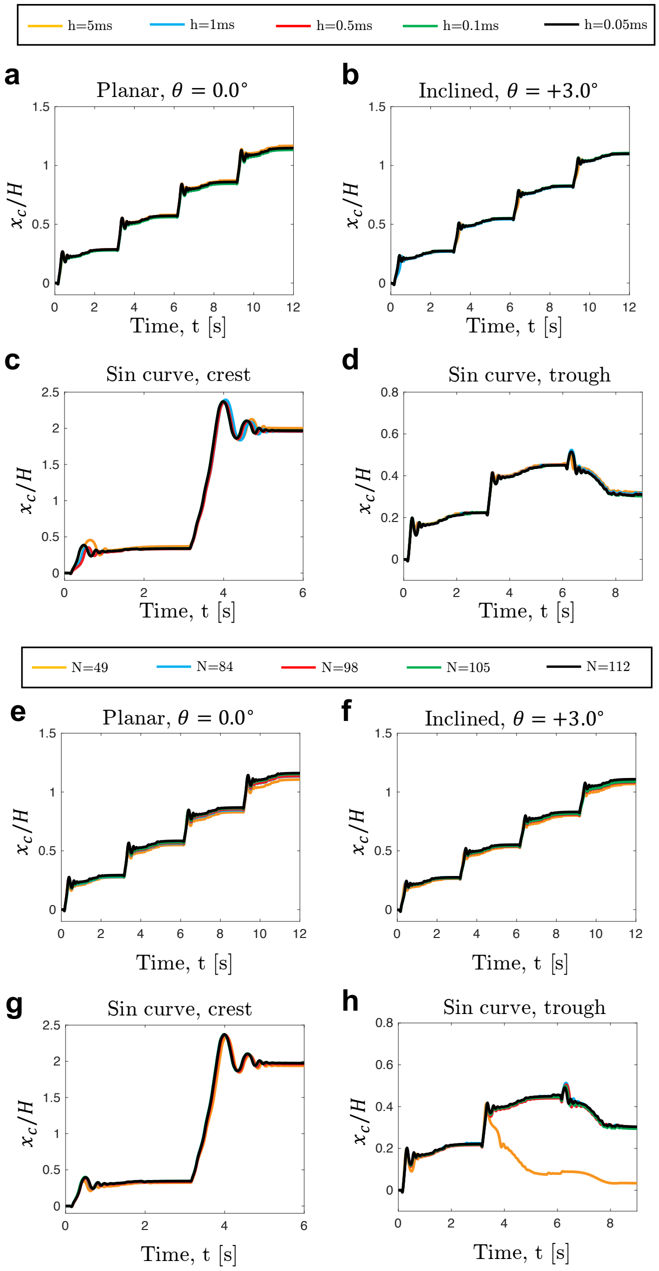

Supplementary Figure 4: Convergence study of rolling robot. **a-d** Convergence study of rolling robot for time discretization (with $N = 84$ fixed). **e-h** Convergence study of rolling robot for space discretization (with $h = 0.1$ms fixed).

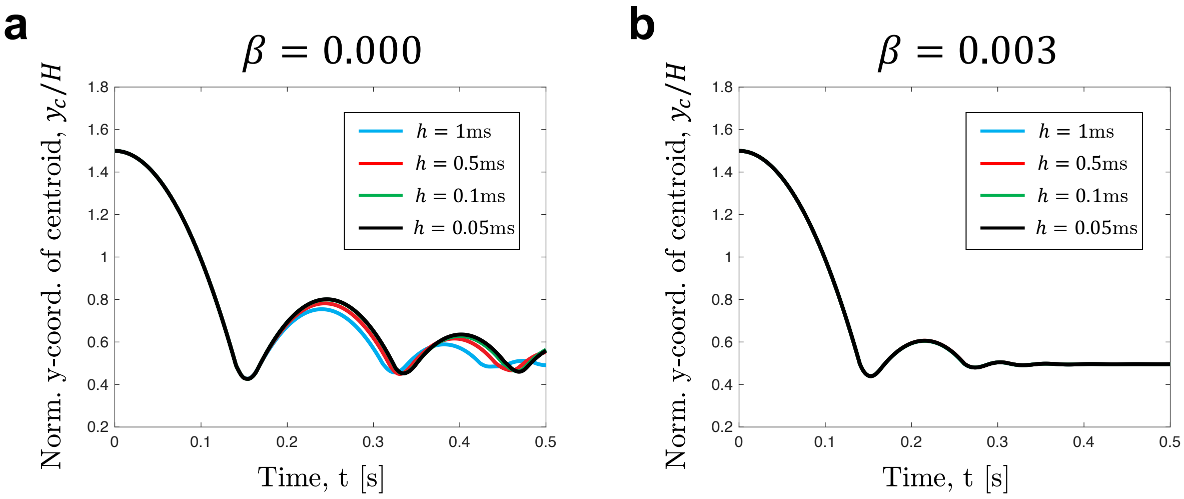

Supplementary Figure 5: Convergence study of rolling robot. Normalized center position y_c/H as a function of time at different values of time step size, h (with number of nodes $N = 84$ fixed) at **a** zero material damping ($\beta = 0$) and **b** non-zero material damping ($\beta = 0.003$). Even though the simulation results vary slightly with time step size with zero material damping, the convergence with time discretization is remarkable with an appropriate amount (see Supplementary Figure 3) of material damping.

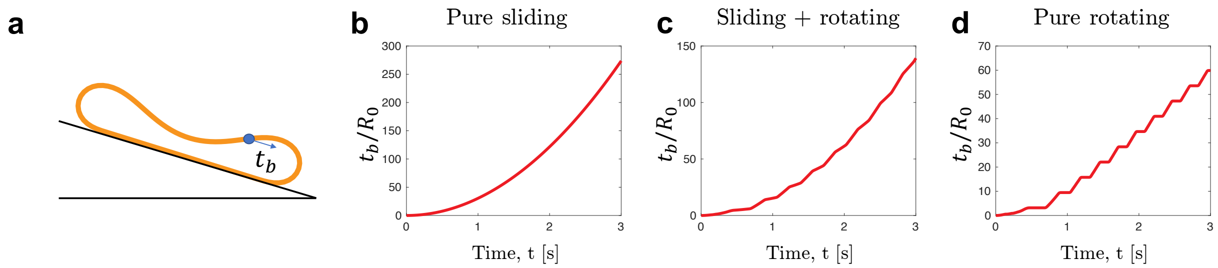

Supplementary Figure 6: Motion patterns of rolling ribbons. **a** Configuration of rolling ribbon with $\Gamma_g = 0.57$. Relative boundary displacement t_b/R_0 as function of time for different motion patterns: **b** pure sliding, **c** combination of sliding and rotating, and **d** pure rotating.

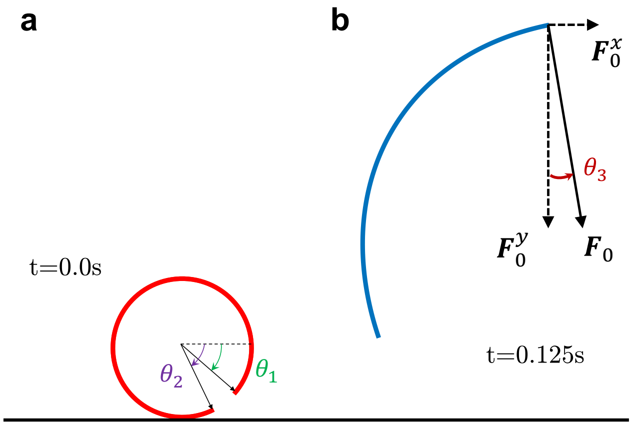

Supplementary Figure 7: Jumper robot. **a** Geometry of jumper robot. **b** Illustration of the drag force from electrical wire.

Supplementary Tables

Supplementary Table 1: Convergence study of rolling robot for time discretization.

Time step size, h	Final normalized centroid x coordinate, x_c/H			
	Planar, $\theta = 0.0°$	Inclined, $\theta = +3.0°$	Sine (crest)	Sine (trough)
$h = 5$ms	1.1660	1.0958	2.0012	0.3206
$h = 1$ms	1.1347	1.0975	1.9549	0.3168
$h = 0.5$ms	1.1356	1.1037	1.9577	0.3122
$h = 0.1$ms	1.1327	1.1050	1.9688	0.3025
$h = 0.05$ms	1.1373	1.0993	1.9672	0.3096

Supplementary Table 2: Convergence study of rolling robot for space discretization.

Number of vertices,	Final normalized centroid x coordinate, x_c/H			
N	Planar, $\theta = 0.0°$	Inclined, $\theta = +3.0°$	Sine (crest)	Sine (trough)
$N = 49$	1.1055	1.0720	1.9289	0.0444
$N = 84$	1.1327	1.1050	1.9688	0.3025
$N = 98$	1.1356	1.0810	1.9598	0.3033
$N = 105$	1.1528	1.0872	1.9740	0.2945
$N = 112$	1.1573	1.1085	1.9738	0.3035

Supplementary Methods

Actuator characterization In this section, we study the mechanical property of a single Shape Memory Alloy (SMA) actuator. We manufactured a single curved SMA actuator sample with the following geometric parameters: arc length $L_s = 33$mm, undeformed curvature $\bar{\kappa}_0 \equiv 1/R_0 = 97.03\text{m}^{-1}$, thickness $h = 1.72$mm, width $w = 19.42$mm. The material density is $\rho = 1920\text{kg}\cdot\text{m}^{-3}$, which is similar to our previous study [1–3].

We employ load-displacement relation to find the Young's modulus of a single SMA actuator for both unactuated and actuated state. In Supplementary Figure 1 **a** (unactuated state) and **b** (actuated state), we use a 20g weight to apply a vertical load at the end of the actuator and evaluate the end displacement. Then we use simulation to find the Young's modulus that best matches the experimental results. In Supplementary Figure 1 **c** and **d**, we plot the best fit configurations of SMA actuator before/after loading. Here, the Young's modulus we found in unactuated state is $E_0 = 3.0$ MPa, and $E_{\text{max}} = 8.04$ MPa in actuated state; these are comparable to the values reported in Ref. [3].

Next, we model the dynamics of a single SMA-based actuator during actuating/cooling process. In Supplementary Figure 2 **a** and **b**, we show the undeformed shape and maximum response shape of SMA-based actuator during heating-cooling process separately. The heating time used in this experiment is 0.25s, and cooling time is 2.75s. We assume that the natural curvature $\bar{\kappa}(t)$

follows a piece-wise function,

$$\bar{\kappa}(t) = \begin{cases} \frac{(n_1-1)t}{t_0}\bar{\kappa}_0 + \bar{\kappa}_0 \text{ when } t < t_0 \\ \frac{(1-n_1)}{1+e^{-\tau(t-\bar{t})}}\bar{\kappa}_0 + n_1\bar{\kappa}_0 \text{ when } t > t_0, \end{cases} \quad (1)$$

and similarly for Young's modulus $E(t)$,

$$E(t) = \begin{cases} \frac{(n_2-1)t}{t_0}E_0 + E_0 \text{ when } t < t_0 \\ \frac{(1-n_2)}{1+e^{-\tau(t-\bar{t})}}E_0 + n_2 E_0 \text{ when } t > t_0, \end{cases} \quad (2)$$

where n_1 and n_2 are the ratios between unactuated state and actuated state, $n_1 = \bar{\kappa}_{\text{min}}/\bar{\kappa}_0$, $n_2 = E_{\text{max}}/E_0$ (the actuated curvature is smaller than unactuated state, while Young's modulus follows an opposite pattern), $t_0 = 0.05\text{s}$, $\bar{t} = 1.4\text{s}$, and $\tau = 3.4\text{s}^{-1}$ are from experimental fitting.

The ratio of Young's modulus, $n_2 = E_{\text{max}}/E_0 = 2.68$, can be easily obtained based on previous loading experiments; another experimentally evaluated parameter, minimum natural curvature, is $\bar{\kappa}_{\text{min}} = 20\text{m}^{-1}$, resulting in $n_1 = 0.21$. We use these fitting parameters to perform this dynamic process in our simulation, and plot the relative beam end position, $X_{\text{end}}/\bar{R}_0$ and $Y_{\text{end}}/\bar{R}_0$, as a function of time during this dynamic process in Supplementary Figure 2 **c**. Then, in Supplementary Figure 2 **d**, we plot these best fitting parameters, e.g. relative Young's modulus E/E_0 and relative natural curvature $\bar{\kappa}/\bar{\kappa}_0$, as a function of time, during the actuating process.

Measurement of Coefficients of Friction The dynamic coefficient of friction μ between the thermally conductive tape and rubber is characterized by giving a slight push to the robot and recording the angle of inclination, θ_0, where it starts to slip down at a steady speed; this method

is inspired by Ref. [3]. The coefficient of friction is prescribed by the relation $\mu = \tan\theta_0$. We also assume that the static frictional coefficient is same as the dynamic coefficient.

Inelastic collision The collision between soft material and rigid platform usually results in partly inelastic collision [4]. Consider the scenario in Supplementary Figure 3 **a** where the rolling robot is dropped from an initial height, L_0. When its two limbs touch the ground, we constraint these two nodes and manually set their normal velocities (relative to the ground) as zeros, to perform the collision. However, as the other parts of rolling robot still have non-zero velocities, the whole structure will be compressed and the kinetic energy will transfer into elastic potential energy. If the viscoelastic behavior pertinent to collision is not considered, the kinetic energy of rolling robot will transfer into potential energy and the structure will rebound to a certain height close to its initial dropped height. Without viscoelasticity, this rebound height is not controllable based on material property.

We add a damping force $\mathbf{F}^{\mathrm{d}} = -(\alpha\mathbb{M} + \beta\mathbb{K})\mathbf{v}$ to the system to model the viscoelastic behavior and inelastic collision of SMA, where $\alpha, \beta \in \mathbb{R}^{+}$, $\mathbb{M}$ is the mass matrix, and $\mathbb{K} = -\frac{\partial}{\partial \mathbf{q}}(\mathbf{F}^{\mathrm{s}} + \mathbf{F}^{\mathrm{b}})$ is the tangent stiffness matrix [4]. The viscosity parameter α is related to external environment damping and can cause momentum dissipation during rigid body motion. As our robot is moving in the air, the effect of environmental damping is negligible, such that $\alpha = 0$. The parameter β is related to material damping and causes energy dissipation only during the deformed process. For rigid body motion, the internal elastic force, $\mathbf{F}^{s} + \mathbf{F}^{b} = \mathbb{K}\mathbf{q}$, is zero; its time derivative (and, therefore, the internal damping force, $\mathbf{F}^{\mathrm{d}} = -\beta\mathbb{K}\mathbf{v}$), should also be zero.

In Supplementary Figure 3 **b**, we plot the normalized y-coordinate of the centroid of the robot, y_c/H, as a function of time, at different values of β. As expected, we find that the rebound height – local maxima in the time series of y_c – decreases with increasing value of β. When $\beta = 1e^{-2}$, there is almost no rebound (i.e. perfectly sticky surface) when the rolling robot is dropped from the height of its own size. We choose $\beta = 3e^{-3}$ that best matches the experimental data. This value is used in all of our simulations on the rolling and jumper robots.

Convergence study Our simulation is robust and shows good convergence with both time and space discretization. In Supplementary Figure 4 **a-d**, we plot the normalized centroid of rolling robot x_c/H, as a function of time, at a fixed number of vertices $N = 84$ and different values of time step size h. Our simulations show good convergence with time for all the following cases: **a** planar motion, **b** inclined surface with $\theta = +3.0°$, **c** crest, and **d** trough. Numerical issues begin to appear beyond $h = 10$ ms. Similarly, we vary number of vertices, N, in Supplementary Figure 4 **e-h** and fix the time step size at $h = 0.1$ ms to show the convergence with space discretization of our simulator. The simulation fails to make quantitative prediction around $N \approx 50$, e.g. see the data corresponding to $N = 49$ in Supplementary Figure 4 **h**. Similar data can be found in Supplementary Table 1 and Supplementary Table 2.

Referring to Supplementary Figure 5, we also note that the convergence with time step size improves when material damping (non-zero β) is introduced. In our simulation scheme, we keep a fixed value of time step size, h. When a node touches the ground, we do not stop integrating the equations of motion; rather, we fix the appropriate degrees of freedom. This introduces some

dependence of the simulation result on time step size especially at zero material damping. Our simulation method can be easily extended to use adaptive time step size for better convergence behavior.

Rolling ribbon In the numerical study of rolling ribbon, the arc length we chose for the circular ribbon is $L_0 = 0.3$m, resulting in $\bar{R}_0 = 0.3/2\pi \approx 0.048$m. The ribbon thickness is $r_0 = 1$mm, poisson ratio $\nu = 0.5$ (incompressible material), material density $\rho = 1237.52$kg/m^3, and we vary the Young's modulus, E, from 1 MPa to 100 MPa to vary the governing dimensionless group Γ_g. The damping parameters are $\alpha = 0$ and $\beta = 1e^{-3}$; however, since we do not quantitatively study the transient dynamics, these parameters do not affect the final deformed configuration and motion patterns.

Here, we show the different motion patterns of rolling ribbon. Consider a circular ribbon with $\Gamma_g = 0.57$ [5] that is moving in a declined surface with $\theta = -17.19°$. Its deformed configuration is shown in Supplementary Figure 6 **a**. The displacement of one boundary node along the declined surface, defined as t_b, is used to quantify the different motion patterns of rolling ribbon. When the relative frictional coefficient $\mu/\tan\theta = 0$, the ribbon slides along the declined surface, without any rotation, resulting in a smooth curve in Supplementary Figure 6 **b**. If the relative frictional coefficient is non zero but smaller than 1, we get a motion that combines sliding and rotating; the zigzag nature of the displacement vs. time data shown in Supplementary Figure 6 **c** arises from the following: the node periodically comes in contact with the surface and, as a result, its velocity slows down. In Supplementary Figure 6 **d**, we show the tangential displacement of boundary node

as a function of time when the ribbon is rotating along the declined surface, without any sliding. From the figure, we can clearly see that the boundary node is totally fixed with the declined surface without any relative displacement once the node touches the ground.

Note that when the ribbon is sliding (pure sliding and the combination of sliding and rotating) along the declined surface, its velocity will continue to increase if environmental damping $\alpha = 0$. In contrast, the speed of pure rotating case remains fixed even when no external damping force is applied into the system.

Geometry of Jumper Robot The geometry of Jumper Robot is an asymmetric circle, as shown in Supplementary Figure 7 **a**. The undeformed radius is $\bar{R}_0 \approx 0.050$m, and other two geometric parameters, $\theta_1 \approx 40^\circ$ and $\theta_2 \approx 60^\circ$, are fitted from experimental image. The material used in Jumper Robot is identical to the one used in the rolling robot, discussed in the previous section.

Also, as shown in Supplementary Figure 7 **b**, we applied a constant force $\mathbf{F}_0$ on the first node on jumper robot over $t_0 \leq t \leq t_1$ (t_0, t_1 are parameters obtained from data fitting) to emulate the presence of the electrical wire. After fitting to experimental data, the horizontal and vertical components of this external force are $F_0^x = 0.05Mg$ and $F_0^y = 0.8Mg$, where M is the total mass of jumper robot. This force is applied at $t_0 = 0.1$s, and ended at $t_1 = 0.15$s.

Supplementary Movie

Supplementary References

1. Huang, X. *et al.* Chasing biomimetic locomotion speeds: Creating untethered soft robots with shape memory alloy actuators. *Sci. Robot.* **3**, eaau7557 (2018).

2. Huang, X. *et al.* Highly dynamic shape memory alloy actuator for fast moving soft robots. *Adv. Mater. Technol.* 1800540 (2019).

3. Goldberg, N. N. *et al.* On planar discrete elastic rod models for the locomotion of soft robots. *Soft Robot.* (2019).

4. Chen, D., Levin, D. I., Matusik, W. & Kaufman, D. M. Dynamics-aware numerical coarsening for fabrication design. *ACM Trans. Graph.* **36**, 84 (2017).

5. Raux, P., Reis, P. M., Bush, J. & Clanet, C. Rolling ribbons. *Phys. Rev. Lett.* **105**, 044301 (2010).
