# Supplementary material for: Dynamic simulation of articulated soft robots
Source: Nat Commun. 2020 May 6;11:2233. doi: 10.1038/s41467-020-15651-9 (PMC7203284; doi:10.1038/s41467-020-15651-9)
Supplement: Supplementary file 4 — Description of Additional Supplementary Files [file 41467_2020_15651_MOESM4_ESM.pdf]

Title: Supplementary Movie 1
Description: Comparison between experiments and simulations.
